# Proposed Methods and Endpoints for Defining and Assessing Adverse Environmental Impact (AEI) on Fish Communities/Populations in Tennessee River Reservoirs

**DOI:** 10.1100/tsw.2002.216

**Published:** 2002-06-07

**Authors:** Gary D. Hickman, Mary L. Brown

**Affiliations:** River System Operations & Environment, Tennessee Valley Authority, 17 Ridgeway Road, Norris, TN 37828, USA

**Keywords:** biocriteria, biological indices, fish community assessment, reservoir fish assemblage index (RFAI), sport fishing index (SFI)

## Abstract

Two multimetric indices have been developed to help address fish community (reservoir fish assemblage index [RFAI]) and individual population quality (sport fishing index [SFI]) in Tennessee River reservoirs. The RFAI, with characteristics similar to the index of biotic integrity (IBI) used in stream fish community determinations, was developed to monitor the existing condition of resident fish communities[1,2,3]. The index, which incorporates standardized electrofishing of littoral areas and experimental gill netting for limnetic bottom-dwelling species, has been used to determine residential fish community response to various anthropogenic impacts in southeastern reservoirs.

The SFI is a multimetric index designed to address the quality of the fishery for individual resident sport fish species in a particular lake or reservoir[4]. The SFI incorporates measures of fish population aspects and angler catch and pressure estimates. This paper proposes 70% of the maximum RFAI score and 10% above the average SFI score for individual species as “screening” endpoints for balanced indigenous populations (BIP) or adverse environmental impact (AEI). Endpoints for these indices indicate: (1) communities/populations are obviously balanced indigenous populations (BIP) indicating no adverse environmental impact (AEI), or are “screened out”; (2) communities/populations are considered to be potentially impacted; and (3) where the resident fish community/population should be considered adversely impacted. Suggestions are also made concerning how examination of individual metric scores can help determine the source or cause of the impact.

## INTRODUCTION

Karr[5] suggested that multimetric indices are robust enough and are more representative of biological responses to anthropogenic influences than traditional water quality monitoring programs. The index of biotic integrity (IBI) originally developed by Karr was used by the Tennessee Valley Authority (TVA) as the basis for development of a fish community quality index in TVA reservoirs. This index was then applied to biomonitoring programs in other geographical regions and aquatic systems[1,2,6,7,8,9,10,11,12]. Jennings[1] first described the multimetric reservoir fish assemblage index (RFAI) as a cost-effective method to address quality of resident fish assemblages as a reflection of environmental quality. The index was further refined[2,3], reducing sampling variability and substituting some metrics that were more reflective of reservoir conditions. Fish community quality is defined as how close resident communities approach the community structure and function anticipated without anthropogenic influence (based on best observed conditions along with professional judgment of biologists familiar with biotic indices and the zoogeography of the Tennessee River). Additional testing of RFAI performance was completed[13] in four reservoirs of both the Catawba and Cumberland River systems to determine the applicability of the index outside the Tennessee River system. Additional minor modifications were made to index metrics. The resulting RFAI was able to distinguish differences between various fish communities in these systems, and results were repeatable. Differences were more difficult to detect within reservoir fish communities, indicating that the biological zone of influence may include large sections of an individual reservoir, including the entire reservoir on smaller impoundments (< 10,000 acres), or that this technique is not sufficiently robust for this application.

Colvin and Vasey[14] first introduced the concept of using multiple metrics in the determination of fishing quality for individual species within a water body. Hickman[4] proposed use of a series of commonly collected population and angler success measures to derive a sport fishing index (SFI) as a measure of recreationally important individual species population quality within a reservoir.

Adverse environmental impact (AEI) endpoints were not adequately developed in section 316(b) of the Clean Water Act of 1972. The definition of AEI required "use of best management practices (BMP) to minimize AEI," but never defined what constituted "AEI" with respect to cooling water intake structure losses. Under 316(a), the thermal effluent endpoint was described as "maintenance of balanced indigenous populations (BIP)," but again did not describe how to determine if BIP existed in the vicinity of a plant. The U.S. Environmental Protection Agency was sued in 1997, requiring a more precise definition of AEI.

The objective of this paper is to suggest potential endpoints for the RFAI and SFI indices. These endpoints suggest (1) where there is no appreciable risk (i.e., no reasonable or significant risk) that resident communities/populations are adversely impacted, (2) levels where adverse impacts are possibly occurring, or (3) communities/populations with obvious unacceptable levels of impact. Suggestions are also made concerning examination of individual metric scores to help determine the source or cause of the impact(s).

## METHODS

Two recent reports[2,3], contain detailed explanations of methods used to arrive at RFAI scores. In general, 15 boat electrofishing samples (each 300 m in length) located proportional to existing shoreline habitat and ten overnight experimental gill net sets (five 6.1 m panels with bar mesh sizes of 2.5, 3.8, 5.1, 6.4, and 7.6 cm) were used to obtain standardized samples of the fish community. Sampling results are compared to reference conditions (i.e., those anticipated from a reservoir in the same physiographic region[15] and reservoir zone in the absence of human-induced impacts other than impoundment and operational characteristics such as winter drawdown). As mentioned previously, reference conditions against which individual samples are compared were derived from best observed conditions of numerous samples (5-year period at several sites in geographically and hydrologically similar reservoirs), with adjustments made by groups of knowledgeable biologists making the criteria more conservative. Scores for individual metrics are assigned using three levels (least degraded-5; intermediate-3; and most degraded-1)[1]. Individual metric scores are then summed to obtain the final RFAI score. RFAI scores from 1993–2000 from upstream and downstream areas in the general vicinity of TVA fossil plants were compared to demonstrate use of these endpoints. Examination of individual metrics was performed to determine potential for plant operation to be contributing to, or causing, adverse impacts.

Hickman[4] described in detail the development and composition of the SFI. The SFI includes information on population parameters and angler success/use routinely collected by many state fishery agencies (Fig. 1). Both population

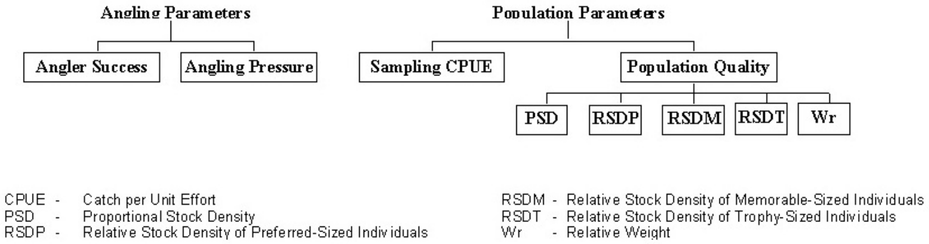

**FIGURE 1.** Parameters used to calculate the sport fishing index.

and angler statistic metrics include quality and quantity aspects. Population quantity measures are simply catch per unit effort by the most appropriate gear type (i.e., electrofishing, gill netting, or trap netting) for the species being addressed. Catch results from only one gear type are used for SFI determination. Population quality measures include size distribution parameters (proportional stock density [PSD] and relative stock density [RSD] of preferred, memorable, and trophy-size groups) and relative weight (Wr) values. Angler catch per hour of intended species addresses the quantity aspect of creel data, and angler use (hours fished for intended species) represents the quality aspect. When creel results were not available, population results were doubled. Though not ideal, this does provide an indication of population quality.

Population and angler results are scored against reference values. Reference values for population quality aspects are those suggested by Gablehouse[16] for maintenance of a balanced multispecies fishery. Reference conditions for both population and angler catch rates were obtained by trisecting historical observed values in Tennessee and Cumberland River reservoirs. As with RFAI, scores were assigned based on the scale of least degraded-5; intermediate-3; and most degraded-1. Metric scores are summed to obtain the SFI score for each important sport fish species.

## RESULTS

Determination of a screening level endpoint (no additional sampling required to demonstrate community AEI or existence of BIP) requires a conservative “no-risk” approach. This was accomplished in three ways. First, RFAI metric scoring criteria were developed on a conservative basis. Reference conditions were based not only on maximum observed values over a large data base, but species expectations were elevated to include any that were historically within the geographic range and were determined to be able to thrive in a reservoir environment.

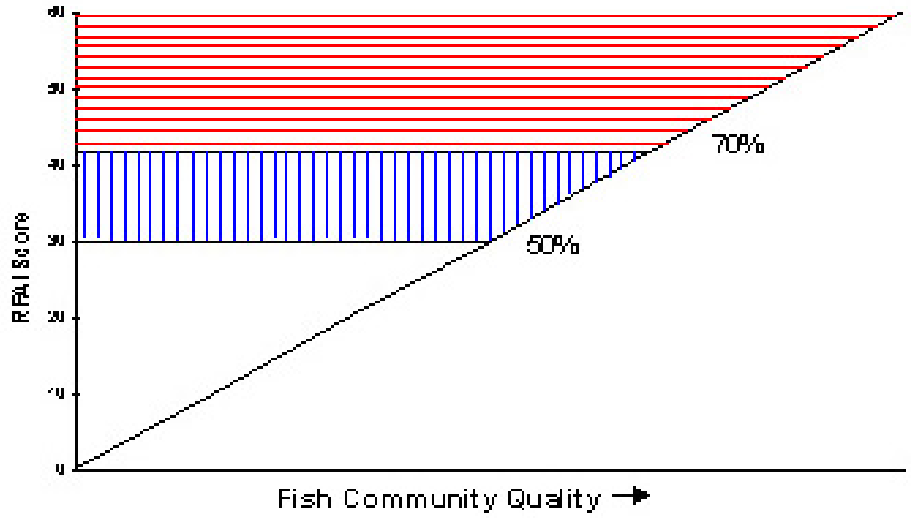

**FIGURE 2.** Proposed RFAI endpoints for determination of adverse environmental impact. If RFAI score is > 70% of attainable score, then the fish community is considered to “screen out” for AEI and have BIP; between 50–70% is considered potentially adversely impacted; and < 50% is considered impacted.

Second, RFAI scores are made even more conservative by removing the calculated sample variability (to prevent “false positives”). This was done by comparison of RFAI scores from 54 paired sample sets (repeat samples within one week) collected over the past seven years. Differences range from 0 to 18 points — the 70$^{th}$ percentile was 6; the 90$^{th}$ percentile was 12. The mean difference between these 54 paired scores was 4.6 points with 95% confidence limits of 3.4 and 5.8. Based on these results, a difference of 6 points or less (±3) was the value selected for defining “similar” scores.

The third conservative level maintains that if more than half of the individual metrics related to impingement/entrainment impacts receive low to moderate scores, then the site fails to screen out. The same requirement is made for thermal impacts and determination of existence of BIP.

To screen out further demonstration of BIP or absence of AEI, it is proposed that the composite RFAI score must exceed 70% (based on conservative measures mentioned above) of the maximum obtainable score of 60 (i.e., RFAI = 42) for that biological zone of the water body, adjusted for defined variability. Fig. 2 graphically shows proposed endpoints. For example, if a site receives an RFAI score of 44 and the mean variability for that reservoir type and zone is ±3, then that site would fail to meet the screening level criteria using the conservative aspect of the variability (+3). It would require a score above 45 to effectively screen out.

**TABLE 1**
**RFAI Metrics Potentially Affected by Impingement/Entrainment and Thermal Impacts**

| Species | Impingement/ Entrainment | Thermal Effects |
|---|---|---|
| Total Species | X | X |
| Average Number of Individuals | X | X |
| Total Centrarchid Species | | |
| Total Benthic Invertivores | X | X |
| Total Intolerant Species | X | X |
| Percent Tolerant | X | X |
| Percent Top Carnivores | X | X |
| Percent Omnivores | X | X |
| Percent Dominance by One Species | X | X |
| Percent Nonnative | X | X |
| Percent Anomalies | | X |
| Largemouth Relative Weight | X | X |

RFAI scores below this screening level do not mean that there is AEI or that BIP do not exist. The endpoint serves as a conservative screening level, i.e., any fish community that receives a score above this level is considered not to have been adversely impacted. RFAI scores below this level would require a more in-depth assessment to determine the likelihood of occurrence of AEI or lack of BIP, and potentially suggest sources of impairment. An inspection of individual RFAI metric results would be an initial step to help identify if plant operation is contributing to lower RFAI scores. Metric scores that will help guide determination of plant operational impacts include looking at what species or groups are missing or underrepresented. When and where do these impacted groups spawn? What are the characteristics of the egg and larval stages? If overall fish densities are low, or if particular groups appear overrepresented, is there attraction to flow or temperature or unique habitat created by operational characteristics? Metrics potentially affected by impingement/entrainment and thermal releases are listed in Table 1. If the RFAI score indicates that the resident fish community has been potentially impacted, impingement and/or entrainment sampling may be required to determine if these potential impact sources are playing major roles in the status of the resident fish community.

A final possible descriptive determination regards whether a resident fish community that receives an RFAI score below a particular trigger level should be labeled as adversely impacted or failing to maintain BIP. This should largely be a site-specific determination with considerable input from the state regulatory agency. An example of an adverse impact trigger level would be a fish community score below 50% of the attainable score of 60 (i.e., RFAI = 30) with adjustment for defined variability (i.e., if variability is ±3, then RFAI = 27). Additional sampling may be necessary to determine responsible agents.

A similar or higher RFAI score at a site downstream of a plant intake/outfall compared to an upstream site has often been used as a basis for determining the presence or absence of impact by fossil plant operation on the resident fish community. Definition of "similar" is integral to accepting the validity of these interpretations between upstream and downstream fish communities. That is, differences between the upstream and downstream fish communities must be more than the natural variation in RFAI scores. If the downstream RFAI score is within 6 points (±3) of the upstream score, the communities are considered similar, and it can be concluded that the plant has had no effect. When an impacted community is suggested by a lower RFAI score, a metric-by-metric examination can be conducted to help determine causes.

A couple of examples from Tennessee Valley reservoirs are used to help visualize how these endpoints would operate. Table 2 shows average RFAI scores from TVA's standardized reservoir monitoring program from both upstream and downstream of some TVA fossil plants from 1993–2000. These are not ideal locations to determine plant operational impacts. Future compliance sampling will be done in immediate upstream/downstream areas beginning in 2001. RFAI values at Bull Run Fossil Plant (BRF) averaged 28 upstream and 37 downstream of the plant. Both values failed the conservative screening criteria, indicating that BIP may not be present and that AEI could be occurring.

**TABLE 2**
**RFAI Scores (1993–2000) in the Vicinity of Various TVA Fossil Plants***

| | | | | Year | | | | | | | | |
|---|---|---|---|---|---|---|---|---|---|---|---|---|
| Plant | Site | Reservoir | Location | 1993 | 1994 | 1995 | 1996 | 1997 | 1998 | 1999 | 2000 | Average |
| | | Tennessee River | | | | | | | | | | |
| | | Upper Mainstream | | | | | | | | | | |
| Watts Bar | Intake | Chickamauga | TRM 529 | 56 | 52 | 44 | 38 | 52 | | 46 | 48 | 48 |
| Watts Bar | Discharge | Watts Bar | TRM 531 | 39 | 43 | | 41 | | 45 | 40 | 45 | 42 |
| Bull Run | Upstream | Melton Hill | CRM 66 | 22 | 28 | | 36 | | 36 | | 32 | 31 |
| Bull Run | Downstream | Melton Hill | CRM 45 | 43 | 43 | | 38 | | 41 | | 47 | 42 |
| Raccoon Mtn | Upstream | Nickajack | TRM 470.2 | 58 | 50 | 54 | | 46 | | 46 | | 51 |
| Raccoon Mtn | Downstream | Nickajack | TRM 425.5 | 49 | 45 | 44 | | 35 | | 34 | | 41 |
| Kingston | Upstream | Watts Bar | CRM 22 | 44 | 40 | | 48 | | 46 | | 42 | 44 |
| Kingston | Downstream | Watts Bar | TRM 560.8 | 53 | 46 | | 42 | | 48 | | 48 | 47 |
| | | Lower Mainstream | | | | | | | | | | |
| Widows Creek | Upstream | Guntersville | TRM 424 | 38 | 42 | | 46 | | 32 | | 32 | 38 |
| Widows Creek | Downstream | Guntersville | TRM 375.2 | 38 | 35 | | 36 | | 30 | | 34 | 35 |
| New Johnsonville | Upstream | Kentucky | TRM 206 | 38 | 34 | 36 | | 38 | | 44 | | 38 |
| New Johnsonville | Downstream | Kentucky | TRM 85 | 44 | 43 | 42 | | 44 | | 38 | | 42 |
| Colbert | Upstream | Pickwick | TRM 259 | 50 | 46 | | 48 | | 42 | | 50 | 47 |
| Colbert | Downstream | Pickwick | TRM 230 | 47 | 47 | | 53 | | 37 | | 47 | 46 |

* Upstream control and downstream impact area sites.

During 2000, the site upstream of BRF scored 32 and the downstream site scored 47 (Table 2). An inspection of individual metric scores revealed no metric received a higher score at the upstream control station than at the downstream station. Two metrics received low scores at both sites including: relative abundance and percent omnivores in both electrofishing and gill netting samples. Four other metrics at the downstream site received moderate scores. These included: total sucker species, total intolerant species, percent tolerant, and percent insectivores. Only four of the nine RFAI metrics potentially related to impingement/entrainment losses received either a low or moderate score, and only five of the 11 metrics potentially related to heated discharge effects received a low or moderate score.

Hickman and Hevel[17] documented a significant inverse relationship between water volume discharged during spring and early summer from upstream Norris Dam and reproductive success of warm water species in Melton Hill Reservoir, and growth of the major piscivore (largemouth bass) in the lake. The periodic releases of hypolimnetic water through Norris Dam can cause considerable fluctuation in daily water temperatures. When this occurs during spawning periods, impacts to the composition of the entire fish community are possible. Metric results tend to support this conclusion as overall Wr of largemouth bass and numbers of fish were depressed. Additionally, percentage of the community comprised of tolerant individuals and omnivores and the number of benthic invertivores were adversely influenced by the daily fluctuations in water temperatures. It is likely that the BRF heated effluent minimally enhances the community downstream of the discharge, as the fish community in this area scored higher than the upstream site during all sample years (1993–2000). The BRF discharge acts to temper the cold water discharged through Norris Dam.

Colbert Fossil Plant (COF) provides an example of a site meeting or approaching the screening level criteria. RFAI scores averaged 48 upstream and 46 downstream of the plant, within the six-point acceptable sample variation, during 1993–2000 (Table 2). The upstream score averaged above the screening criteria and did so four out of the five years this site was sampled. The downstream site average was slightly above the screening level and scored above screening out in four of the five years. This was the only plant out of the seven Tennessee River plant sites sampled where both upstream and downstream sites exceeded the conservative screening level, indicating that resident fish communities at these locations are not adversely impacted.

The SFI provides a mechanism of screening for an individual species population within a reservoir. Fig. 3 provides an example of SFI results for black bass in Tennessee and Cumberland River reservoirs during 2000. It is proposed that any individual species population successfully screens out of additional BIP

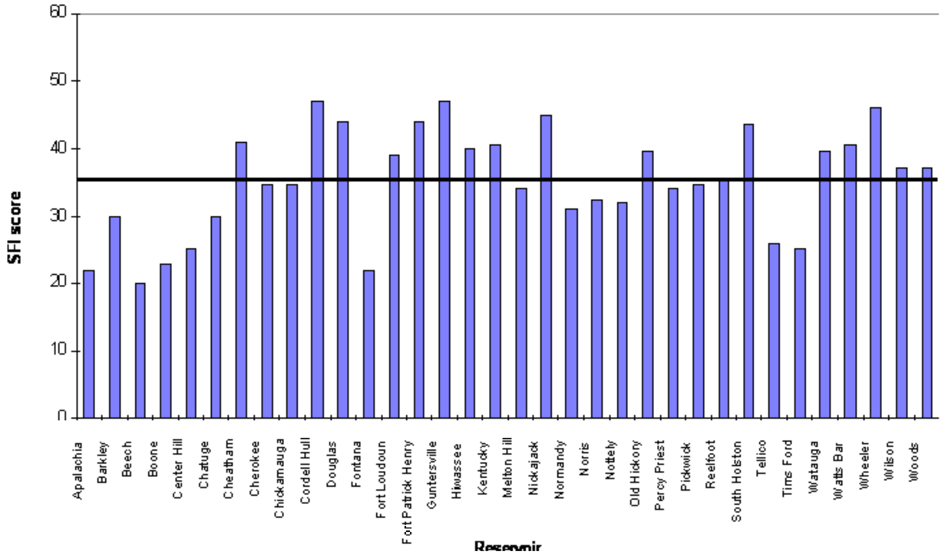

**FIGURE 3.** Black bass SFI scores for 2000. Line indicates overall average**.**

or AEI determinations if the SFI score is 10% above average for all reservoirs with SFI data for that particular year. Use of this endpoint requires a complete range of population quality (from excellent to poor).

A species population score more than 10% below average is the trigger point indicating that AEI may be occurring with regard to that species population. If the SFI score does suggest adverse impacts, inspection of individual metric scores may give insight on the potential of plant-induced impacts.

Melton Hill Reservoir SFI results, based on population data only, as no angler catch or pressure information were available for 2000, indicate a striped bass/hybrid population well above the 10% above average screening level (Table 3). Channel catfish and largemouth bass populations were below the upper screening level, but were not low enough to indicate impacted populations. Densities of smallmouth bass and spotted bass were too low to develop accurate length frequency or relative health analyses. The bluegill population was dominated by young individuals with a PSD of only 8.9 and no fish of preferred, memorable, or trophy size. The Wr value of 75 indicates that resident bluegill are well below anticipated weights per unit length. Catch rate received a moderate score (see Table 4).

The channel catfish population in Melton Hill received a high PSD score indicating a lack of sufficient recruitment. A moderate number of preferred-size fish were present, but no memorable or trophy-size individuals. The Wr was slightly low and the catch rate was moderate. The aforementioned conditions

**TABLE 3**
**Sport Fishing Index (SFI) Scores for Representative Important Species During 2002**

| Reservoir | Black Bass | Bluegill | Channel Catfish | Crappie | Largemouth Bass | Sauger | Smallmouth Bass | Spotted Bass | Striped Bass or Hybrids | Walleye | White Bass |
|---|---|---|---|---|---|---|---|---|---|---|---|
| Appalachia | 22* | 20* | 20* | | 20* | | | 20* | | 20 | |
| Barkley | 30 | 42 | 20 | 43 | 30 | | 20* | 20* | | | 20 |
| Beech | 20 | 20* | 34* | | 32* | | | | | | |
| Boone | 23 | | | | 28* | | 46* | | | | |
| Center Hill | 25 | 30 | 20 | 20 | 20 | | 20 | | | 40 | |
| Chatuge | 30* | 26* | 26* | | 30* | | 20* | 30* | 30* | 36 | 48 |
| Cheatham | 41 | 32* | | | 44* | | | 32*8 | | | |
| Cherokee | 35 | 26 | 23 | 41 | 32 | 24* | 27 | 22 | 52 | 25 | 30 |
| Chickamauga | 35 | 33 | 29 | 31 | 32 | 39 | 22 | 40* | 30 | 20 | 30 |
| Cordell Hull | 47 | | | | 34* | | 26* | | | | |
| Douglas | 44 | 30 | 20 | 28 | 44 | 20 | 20 | | | 20 | 20 |
| Fontana | 22* | 22* | 20* | | 32* | | 26* | 20* | 48* | 46 | |
| Fort Loudoun | 39 | 20* | 20* | | 36* | 42* | 32* | 26* | 46* | | |
| Fort Patrick Henry | 44* | | | | 24* | | 42* | | | | |
| Guntersville | 47 | 22* | 24* | | 32* | 26* | 20* | 34* | 20* | 20 | |
| Hiwassee | 40* | 24* | 20* | | 28* | | 24* | 32* | 20* | 48 | |
| Kentucky | 41 | 44 | 50 | 47 | 32 | 40 | 32 | 32* | 20 | | 30 |
| Melton Hill | 34 | 20* | 24* | | 30* | 20* | 20* | 20* | 54 | 20 | |
| Nickajack | 45 | | | | 50* | | | 52* | | | |
| Normandy | 31 | 40 | 20 | 20 | 32 | 20* | 25 | 32* | 20* | 20 | |
| Norris | 33 | 30 | 20 | 20 | 26 | 20 | 30 | 40* | 24 | 24 | |
| Nottely | 32* | 30* | 20* | | 30* | | 20* | 32* | 42* | | 40* |
| Old Hickory | 40 | 31 | 20 | 30 | 37 | 20 | | | 20 | | 20 |
| Percy Priest | 34 | 35 | 20 | 30 | 34 | | 20 | 40* | 30 | | 20 |
| Pickwick | 35 | 33 | 20 | 21 | 27 | 40 | 37 | 24* | 20 | | 40 |
| Reelfoot | 36 | 49 | 20 | 60 | 33 | | | | | | |
| South Holston | 44 | 26 | 20 | 20 | 36 | | 55 | | 20* | 32 | |
| Tellico | 26* | | | | 28* | | 20* | 32* | | | |
| Tims Ford | 25 | 40* | 20* | | 32* | 20* | 22* | 24* | 24* | 20 | |
| Watauga | 40 | 27 | 20 | 30 | 33 | | 52 | 20* | | 41 | |
| Watts Bar | 41 | 27 | 22 | 37 | 43 | 34 | 28* | 38* | 39 | | 25 |
| Wheeler | 46 | 24* | 20* | | 28* | 20* | 44* | 20* | 22* | | |
| Wilson | 37 | 22* | | | 52* | | 56* | 20* | 20* | | |
| Woods | 37 | 42 | 20 | 20 | 37 | | 20* | | | | |
| Average | 35.3 | 29.9 | 22.7 | 31.1 | 32.9 | 27.5 | 29.5 | 29.3 | 30.1 | 28.8 | 29.4 |

resulting from the influence of Norris Dam periodic releases are revealed by these metric scores for Melton Hill Reservoir[17]. Large fluctuations in water temperatures during spawning in most years lead to large differences in year class strength as shown by these species. Striped bass and hybrids are stocked into the reservoir to maintain these populations. Only largemouth bass seem capable of maintaining an average population in the reservoir. SFI determinations are reservoir-wide and cannot be used in upstream/downstream comparisons. However, as mentioned previously, the fish community below the plant thermal

**TABLE 4**
**Sport Fishing Index Results for Melton Hill and Pickwick Reservoirs**

| lton Hill | PSD | | RSDP | | RSDM | | RSDT | | Wr | | Catch Rate | | Creel Catch | | Pressure | | Bait Bite | | |
|---|---|---|---|---|---|---|---|---|---|---|---|---|---|---|---|---|---|---|---|
| ecies | (a) | (b) | (a) | (b) | (a) | (b) | (a) | (b) | (a) | (b) | (a) | (b) | (a) | (b) | (a) | (b) | (a) | (b) | SFI |
| ack bass | 37.2 | 2* | 9.0 | 2 | 1.3 | 2 | 0.0 | 1 | 89.4 | 2 | 15.6 | 5 | | | | | 2.0 | 20 | 34 |
| uegill | 8.9 | 2 | 0.0 | 2 | 0.0 | 2 | 0.0 | 2 | 75.5 | 2 | 61.3 | 10 | | | | | | | 20 |
| annel catfish | 83.3 | 2 | 4.2 | 4 | 0.0 | 2 | 0.0 | 2 | 87.9 | 4 | 1.0 | 10 | | | | | | | 24 |
| brid Striped × White ss | 36.4 | 4 | 15.2 | 6 | 9.1 | 6 | 0.0 | 2 | 92.9 | 6 | 1.4 | 30 | | | | | | | 54 |
| rgemouth bass | 35.7 | 4 | 5.7 | 4 | 1.4 | 4 | 0.0 | 2 | 90.7 | 6 | 14.0 | 10 | | | | | | | 30 |
| uger | 0.0 | 2 | 0.0 | 2 | 0.0 | 2 | 0.0 | 2 | 0.0 | 2 | 0.1 | 10 | | | | | | | 20 |
| nallmouth bass | 0.0 | 2 | 0.0 | 2 | 0.0 | 2 | 0.0 | 2 | 0.0 | 2 | 0.2 | 10 | | | | | | | 20 |
| otted bass | 0.0 | 2 | 0.0 | 2 | 0.0 | 2 | 0.0 | 2 | 0.0 | 2 | 1.4 | 10 | | | | | | | 20 |
| alleye | 0.0 | 2 | 0.0 | 2 | 0.0 | 2 | 0.0 | 2 | 0.0 | 2 | 0.2 | 10 | | | | | | | 20 |

| ckwick | PSD | | RSDP | | RSDM | | RSDT | | Wr | | Catch Rate | | Creel Catch | | Pressure | | Bait Bite | | |
|---|---|---|---|---|---|---|---|---|---|---|---|---|---|---|---|---|---|---|---|
| ecies | (a) | (b) | (a) | (b) | (a) | (b) | (a) | (b) | (a) | (b) | (a) | (b) | (a) | (b) | (a) | (b) | (a) | (b) | SFI |
| ack bass | 62.5 | 3 | 17.1 | 3 | 2.3 | 2 | 0.0 | 1 | 96.0 | 3 | 58.7 | 5 | 0.8 | 7.5 | 7.0 | 5 | 2.0 | 5 | 34.5 |
| uegill | 67.0 | 3 | 2.0 | 2 | 0.0 | 1 | 0.0 | 1 | 0.0 | 1 | 66.7 | 15 | 1.4 | 5 | 0.5 | 5 | | | 33 |
| appie | 0.0 | 1 | 0.0 | 1 | 0.0 | 1 | 0.0 | 1 | 80.9 | 2 | 2.2 | 5 | 0.4 | 5 | 0.5 | 5 | | | 21 |
| rgemouth bass | 61.2 | 3 | 17.9 | 3 | 3.0 | 2 | 0.0 | 1 | 95.3 | 3 | 44.7 | 5 | 0.2 | 5 | 7.0 | 5 | | | 27 |
| uger | | | | | | | | | | | | | 1.3 | 30 | 0.3 | 10 | | | 40 |
| nallmouth bass | 70.0 | 3 | 17.5 | 3 | 2.5 | 2 | 0.0 | 1 | 93.2 | 3 | 8.7 | 10 | 0.2 | 10 | 7.0 | 5 | | | 37 |
| otted bass | 0.0 | 2 | 0.0 | 2 | 0.0 | 2 | 0.0 | 2 | 92.5 | 6 | 5.3 | 10 | | | | | | | 24 |
| nite bass | | | | | | | | | | | | | 4.0 | 30 | 0.1 | 10 | | | 40 |

(a) - Attained Value  RSDM - Relative Stock Density of Memorable-Sized Individuals
(b) - Assigned Score Based on Attained Value  RSDT - Relative Stock Density of Trophy-Sized Individuals
PSD - Proportional Stock Density  SFI - Sport Fishing Index
RSDP - Relative Stock Density of Preferred-Sized Individuals  Wr - Relative Weight

*No creel information available; therefore population metric scores were doubled to obtain comparable SFI score.

discharge is superior to those found upstream of the plant, suggesting a positive influence. However, the influence is not substantial enough to improve all sport fish populations on a reservoir-wide basis.

SFI results indicate that Pickwick Reservoir provides populations of bluegill, sauger, smallmouth bass, and white bass that exceed the 10% above average screening level. (Table 3). However, crappie and spotted bass did not meet the 10% above or below average screening criteria, suggesting that these populations may be failing to reach their potential. The Pickwick spotted bass and crappie populations received low or moderate scores from all aspects. Spotted bass habitat is limited in Pickwick due to limited availability of their preferred steep, rocky banks and relatively low nutrient levels, but Pickwick does maintain adequate habitat capable of supporting a reasonable crappie population. Under these circumstances, additional sampling could be necessary to demonstrate whether or not plant operation is impacting the crappie population in Pickwick Reservoir.

## CONCLUSIONS

RFAI and SFI indices can be used to define various levels of fish community/population quality within a reservoir. A “no-risk” screening level for demonstration of BIP, or no AEI, when attained RFAI scores exceed 70% of the maximum score of 60 (RFAI = 42), appears suitable to protect resident fish communities. The screening level endpoint must be adjusted for defined variability in the index score for that reservoir type and zone (i.e., with variability $\pm$3, RFAI = 45 would screen out). Due to the conservative manner in which index reference conditions are developed, this level minimizes the potential of screening out a fish community that is adversely impacted. If a fish community fails to exceed the RFAI screening level score, it does not mean that the community is adversely impacted, just that additional information is necessary to make that determination. A possible endpoint where the resident fish community may be considered to be adversely impacted would be if the RFAI score fell below 50% of the maximum score, adjusted for average variability (i.e., with variability $\pm$3, RFAI = 27).

RFAI scores were successfully used to describe fish community status in reservoirs with fossil and nuclear plant intake and thermal discharges using upstream control and downstream potentially impacted areas. Some fish communities failed to attain the conservative screening level; some were below a proposed endpoint, suggesting that they were adversely impacted; and a couple of sites did meet the screening level criteria.

Two examples used to demonstrate how the screening process works included one incident where the fish community and individual sport fish populations failed to screen out and one where most or all screen-out criteria were met. The Melton Hill Reservoir fish community in the vicinity of BRF, the upstream (RFAI = 32) site failed the screening level criteria (RFAI = 45) (Table 5). However, inspection of individual metric results and knowledge of other potential influencing factors led to the determination that plant operation was actually having a positive impact on the downstream population, although this impact was not sufficient to override the negative impacts of upstream hypolimnetic reservoir releases.

RFAI scores for Pickwick Reservoir in the vicinity of COF exceeded the screening level criteria. The upstream control site RFAI score averaged 47 and exceeded the screening level trigger of 45 in four out of the five years sampled. The downstream site averaged 46, just above the necessary screening score of 45, and attained the screening level in four of the five sample years.

Upstream/downstream scores were within the six-point acceptable sample variation, indicating no appreciable difference in fish communities residing in these areas.

The SFI screening criteria of maintaining average or above-average individual sport fish populations also appears useful. Inspection of metric scores proved insight could be gained into possible factors or conditions that might be limiting a particular population. Again using Melton Hill and Pickwick

**TABLE 5**
**Individual RFAI Metric Results From Melton Hill Reservoir Samples in the Vicinity of Bull Run Steam Plant, Fall 2000**

| RFAI Metrics | Upstream | Downstream |
|---|---|---|
| Total Species | 3 | 5 |
| Total Centrarchid Species | 3 | 5 |
| Total Sucker Species | 3 | 3 |
| Total Intolerant Species | 3 | 3 |
| Percent Tolerant (EF) | 1 | 1.5 |
| Percent Tolerant (XGN) | | 2.5 |
| Percent Dominance by One Species (EF) | 3 | 2.5 |
| Percent Dominance by One Species (XGN) | | 2.5 |
| Number of Piscivore Species | 3 | 5 |
| Percent Omnivores (EF) | 1 | 1.5 |
| Percent Omnivores (XGN) | | 0.5 |
| Percent Insectivores (EF) | 3 | 1.5 |
| Percent Insectivores (XGN) | | 2.5 |
| Number of Lithophilic Spawning Species | 3 | 5 |
| Average Number of Individuals (EF) | 1 | 0.5 |
| Average Number of Individuals (XGN) | | 0.5 |
| Percent Anomalies | 5 | 5 |
| **Score** | **32** | **47** |

EF = electrofishing, XGN = gill netting.

Reservoirs as examples, some individual species populations screened out in both reservoirs, and some required an in-depth look at metric scores to determine possible sources of stress on these populations.

In summary, screening level endpoints would be very helpful for both regulators and utilities alike. A series of endpoints for RFAI and SFI multimetric indices can be used to determine if existing fish communities/populations are healthy and whether or not they remain that way after plant operation begins. Establishment of such endpoints, based upon sound indices, could reduce the amount of extensive sampling necessary without jeopardizing the well-being of the resident fish community or individual sport fish populations. In cases that meet the conservative screening level, periodic low-intensity fish community/population monitoring would be sufficient to determine if problem situations develop.

## BIOSKETCHES

**Gary D. Hickman** is a Principal Environmental Scientist with the Tennessee Valley Authority in Norris, Tennessee. He received B.S. and M.S. degrees from the University of Arkansas and is a Certified Fisheries Scientist by the American Fisheries Society.

**Mary L. Brown** is an Environmental Scientist with Westaff Technical (TVA contract), Norris, Tennessee. She received a B.S. degree from the University of Tennessee.
